# Network neurosurgery

**DOI:** 10.1186/s41016-023-00317-4

**Published:** 2023-02-02

**Authors:** Jizong Zhao

**Affiliations:** 1 China National Clinical Research Center for Neurological Diseases, Beijing, China; 2 National Center for Neurological Disorders, Beijing, China; 3 Department of Neurosurgery, Beijing Tiantan Hospital, Capital Medical University, Beijing, China

Entering the twenty-first century, the centenary neurosurgery has entered the era of network neurosurgery, along with the development of brain research [1–3], the digitization of medical imaging, and the establishment of hybrid operating rooms.

Network neurosurgery is based on the topological characteristics of the brain networks, using multimodal intelligent neurological imaging and minimally invasive surgical techniques. In the process of diagnosing and treating neurological diseases, network neurosurgery can protect and remodel the patient’s functional brain networks and thus improve the level of diagnosis and treatment of refractory neurological disorders. Network neurosurgery will be the nexus for translational research in the field of human brain science.

## Three historical stages of the development of neurosurgery in the last hundred years

In the early twentieth century, neurosurgery gradually became an independent clinical specialty by a deep understanding of brain neurophysiology and functional localization. The centenary history of neurosurgery can be divided into three stages: classical neurosurgery, microneurosurgery, and minimally invasive neurosurgery. The three dimensions of brain anatomy and cognitive discovery, leap development of biomedical imaging, and the update of surgical instruments are the driving force of the continuous development of neurosurgery.

### The stage of classical neurosurgery

In the late nineteenth century, surgeons began to engage in the surgical treatment of intracranial tumors, brain abscesses, epilepsy, spinal cord compression, and trigeminal neuralgia. In 1870, Fritsch and Hitzig firstly proved the functional localization of the parietal cortex. Subsequently, Flechsig mapped the functional areas of movement, sensation, and vision in human brains and established the examination methods of the nervous system. The diagnostic techniques of pneumoencephalography (1917), cerebral angiography (1927), electroencephalogram (EEG) (1929), and other specialized methods detecting the location of intracranial lesions have laid the foundation for neurosurgery. In 1919, Cushing (1869–1939), a neurosurgeon, proposed and established the neurosurgery. During this stage, the neurosurgery solved the patients’ increased intracranial pressure and neurological deficits caused by intracranial lesions.

### The stage of microneurosurgery

From the 1950s to the late twentieth century, the successive appearance of computerized tomography (CT), magnetic resonance imaging (MRI), and digital subtraction angiography (DSA) provided reliable imaging evidence for early detection and accurate localization of intracranial lesions. The surgical microscope and microsurgical instruments, such as high-speed cranial drill, controllable operating table and skull frame, automatic retractor, ultrasonic suction, and bipolar coagulation, solved the problems of lighting, narrow field, and hemostasis that interfered with the process of neurosurgery. The new procedure of microsurgery, especially the transcranial approach, can accurately remove the brain lesions while effectively protecting the normal brain structure.

### The stage of minimally invasive neurosurgery

The minimally invasive neurosurgery was capable of early and accurate diagnosing neurological disorders and locating the functional areas based on modern diagnostics imaging. The neurosurgical equipment of navigation systems, neuro-endoscopy, cerebral blood flow, and electrophysiological monitoring have provided more reliable guarantees for the accurate detection of lesions and avoidance of neurological impairment during surgery. Endovascular interventional techniques and stereotactic radiosurgery have become new modalities for minimally invasive neurosurgery.

## Neurosurgery has entered the era of networks neurosurgery in the twenty-first century

Entering the twenty-first century, the research boom of brain science, the digitization of medical imaging, and the emergence of modern technology in hybrid surgery have promoted neurosurgery to the era of networks neurosurgery.

The human brain is the most complex object in the known universe. Understanding the structure and function of the brain is the most challenging frontier scientific problem in the twenty-first century. Brain research has important clinical significance for the effective diagnosis and treatment of brain disorders. Brain science research reveals the theory of cerebral networks and promotes neurosurgery in the era of networks neurosurgery.

Modern digital neuroimaging, such as functional MRI (fMRI), DSA, single photon emission computed tomography (SPECT), positron emission tomography (PET), and other multimodal neuroimaging technologies, has achieved the leap from anatomical neuroimaging to functional neuroimaging. The structural brain imaging and functional networks have laid a solid foundation for networks neurosurgery.

The modern neurosurgical hybrid operating room is equipped with intraoperative MRI and cerebral angiography equipment, which provides real-time imaging data and thereby guides the operation. Intraoperative waken-up anesthesia, frameless stereotaxic robots, intraoperative neurophysiological monitoring (IONM), and other technologies have established a technological platform for networks neurosurgery.

In the procedure of networks neurosurgery, the neural network and its relay station can be precisely positioned. The intraoperative resection of intracranial lesions and implantation of electrodes are accomplished with neuroengineering, nanotechnology, brain-computer interface, and other technologies. Networks neurosurgery not only treats brain tumors and neurovascular diseases but also opens new avenues for chronic pain (facial, somatic, afferent sensory disorders, phantom limbs), movement disorders (Parkinson’s disease, dystonia), epilepsy, mental disorders, hearing impairment, vision loss, and other refractory neurological disorders.

## Prospects of networks neurosurgery

### Protection of key hubs in brain networks

The vast majority of brain hubs in the human brain functional networks have only a few connectivities, while a few hubs have a great many connectivities, known as the key hubs of the brain networks. The structure and function of the networks will be seriously affected if the key hubs were damaged [2]. Exploration of the location and function of the key hubs, including language area [3], visual area [4], and intrinsic functional connectivity [5], is important for networks neurosurgery. Brain function has great potential for remodeling and recovery [6]. If the key hubs were not damaged during surgery, the postoperative short-term neurological deficits can be expected to recover. Therefore, it is important to fully understand the topological characteristics of the brain networks for networks neurosurgery. By finding the key hubs of the networks and accurately selecting the surgical approach, it is feasible to include patients who are expected to recover in the short term and subsequently improve the long-term prognosis.

### Protection of the blood supply areas of key hubs in brain networks

The blood supply and the structure of brain networks are strongly linked to functional connectivity [7–10]. Although the cognitive-related functional areas are not involved in many cerebrovascular diseases, they may still promote cognitive impairment in the long-term follow-up. Concerns regarding the well-known structures related to brain functions, as well as the cognitive impairment that may be caused by the destruction of blood supply to key hubs of the brain networks, are required in the process of applying the concept of brain network protection to the surgery. Therefore, a preoperative comprehensive evaluation of brain function, blood vessels, and networks are warranted, and minimally hazardous management is selected consequently.

### Revascularization and functional remodeling of brain networks

Revascularization contributes to the recovery of brain functional networks in patients with ischemic stroke. Chronic cerebral hypoperfusion affects the structure and function of fiber tracts in adult patients with moyamoya disease (MMD) [11], and the cognitive impairment was shown to be associated with low-frequency fluctuations in the resting-state fMRI [12, 13]. In addition, the asymptomatic manifestation of patients with carotid artery stenosis may be related to the compensatory functional connection in the contralateral cerebral hemisphere [14].

The prediction of recovery in brain network connectivity is available after the revascularization by brain network analysis, which may contribute to predicting the selection of surgical management. Prospectively, the preoperative evaluation will no longer be limited to the degree of ischemia in the management of cerebrovascular diseases. The surgical indications and prediction of surgical outcomes are determined regarding the status of brain network connectivity.

### Mechanism research on the consciousness and cognitive impairment

The generation and regulation of consciousness and cognition exist at the level of neural circuit. The cerebral cortex plays a central role in consciousness and cognitive functions. There may be complex neuronal subsets in the single brain region. Central circuit is the core circuit that generates, maintains, and regulates the consciousness. The core structure of central thalamus is a key target for deep brain stimulation (DBS) in consciousness disorders. The functional connectivity and neural oscillatory characteristics of the central thalamocortical circuit are strongly associated with loss and recovery of consciousness. Different frequencies of electrical stimulation can awaken the state of loss of consciousness. Therefore, it is a key way to resolve individual differences, increase the rate of awakening, and shorten the awakening duration, by developing an adaptive approach of closed-loop neuromodulation based on the regulatory mechanisms and theories and forming an effective clinical practice for DBS awakening in consciousness disorders. Besides, it provides novel ideas for analyzing the “Principles of Brain Cognition and Neural Mechanisms of Consciousness.”

### Neuromodulation technology to analyze the treatment mechanism of DBS

By directly measuring the pathological brain activity and providing adjustable stimulation, DBS is feasible to treat neurological and psychiatric disorders related to abnormal circuit function. The brain electrodes and pacemaker [15, 16], which are highly compatible with MRI, can display the effects of local dysfunction and interventional management on the whole brain networks in real time under the working state of DBS. The technology can also analyze the mechanisms of DBS treatment in various neurological diseases, including chronic pain (facial, somatic, deafferentation, phantom limb symptoms), movement disorders (Parkinson’s disease, dystonia, Tourette syndrome), epilepsy, and psychiatric disorders. Hence, it is of great significance to understand the effect of DBS on brain neuromodulation.

### Clinical practice of brain-computer interface

Brain-computer interface (BCI) establishes a new communication and control channel between the brain and the external environment that is independent of peripheral nerves and muscles. Thus, it is practicable to accomplish the direct interaction of the brain and external devices. BCI can be used as an auxiliary communication and control method for patients with severe movement disorders. By utilizing the signals of the motor cortex, patients can precisely control the external robotic arm and manipulator and restore motor dysfunction. Obtaining and analyzing the EEG signals of patients with chronic consciousness disorders through BCI equipment, the diagnosis and assessment, prognosis evaluation, and even communication of consciousness disorders can be achieved. Further clinical research on neurodegenerative diseases (Alzheimer’s disease and Parkinson’s disease, etc.) is warranted.

In the twenty-first century, brain research has advanced by leaps and bounds, targeting the network neurosurgery. Therefore, actively and rationally constructing the new discipline is an arduous task for the neurosurgery community [17].

## References

[1] Fox MD (2018). Mapping symptom brain network with human connectome. N Engl J Med.

[2] Power JD, Schlaggar BL, Lessov-Schlaggar CN (2013). Evidence for hubs in human functional brain networks. Neuron.

[3] Xu Y, He Y, Bi Y (2017). A tri-network model of human semantic processing. Front Psychol.

[4] Popham SF (2021). Visual and linguistic semantic representationa are aligned at the border of human visual cortex. Nature Neuroscience.

[5] Buckner RL, Sepulcre J, Talukdar T, et al. Cortical hubs revealed by intrinsic functional connectivity: mapping, assessment of stability, and relation to Alzheimer’s disease.The Journal of neuroscience: the official journal of the Society for Neuroscience 2009;29(6):1860–73.doi:10.1523/jneurosci.5062-08.2009 [published Online First:2009/02/13] 10.1523/JNEUROSCI.5062-08.2009 PMC2750039 19211893

[6] Jiang T, Wang Y, Fang S. Comprehensive delineation of the topological properties and protection mechanisms of motor function networks. Chinese J Neurosurg. 2020;36(2):109–11. 10.3760/cma.j.issn.1001-2346.2020.02.001.

[7] Bullmore E, Sporns O (2012). The economy of brain network organization. Nat Rev Neurosci.

[8] Bálint V, Mustafa C, Alexander K (2011). Quantifying the link between anatomical connectivity, gray matter volume and regional cerebral blood flow: an integrative MRI study. PLoS ONE.

[9] Sepulcre J, Liu H, Talukdar T (2010). The organization of local and distant functional connectivity in the human brain. PLoS Comput Biol.

[10] Xia L, Zou Q, He Y (2013). Coupling of functional connectivity and regional cerebral blood flow reveals a physiological basis for network hubs of the human brain. Proc Natl Acad Sci.

[11] Kazumata K, Tha KK, Narita H (2015). Chronic ischemia alters brain microstructural integrity and cognitive performance in adult moyamoya disease. Stroke.

[12] Lei Y, Li Y, Ni W (2014). Spontaneous brain activity in adult patients with moyamoya disease: a resting-state fMRI study. Brain Res.

[13] Lei Y, Song B, Chen L (2020). Reconfigured functional network dynamics in adult moyamoya disease: a resting-state fMRI study. Brain Imaging Behav.

[14] He S, Liu Z, Xu Z (2020). Brain functional network in chronic asymptomatic carotid artery stenosis and occlusion: changes and compensation. Neural Plast.

[15] Siyuan Zhao, Gen Li, Chuanjun Tong, et al: Full activation pattern mapping by simultaneous deep brain stimulation and fMRI with graphene fiber electrodes.Nature Communications volume11, Article number:1788 (2020) Cite this article 10.1038/s41467-020-15570-9 PMC7156737 32286290

[16] Zhang F, Jiang C, Li Y (2021). Investigation of artifacts and optimization in proton resonance frequency thermometry towards heating risk monitoring of implantable medical devices in magnetic resonance imaging. IEEE Trans Biomed Eng.

[17] Zhao J. Network neurosurgery: a look into the future. Sci Technol Rev. 2020;38(10):63–65.

